# A random mutagenesis screen enriched for missense mutations in bacterial effector proteins

**DOI:** 10.1093/g3journal/jkae158

**Published:** 2024-07-19

**Authors:** Malene L Urbanus, Thomas M Zheng, Anna N Khusnutdinova, Doreen Banh, Harley O’Connor Mount, Alind Gupta, Peter J Stogios, Alexei Savchenko, Ralph R Isberg, Alexander F Yakunin, Alexander W Ensminger

**Affiliations:** Department of Biochemistry, University of Toronto, Toronto, ON M5G 1M1, Canada; Department of Biochemistry, University of Toronto, Toronto, ON M5G 1M1, Canada; Department of Chemical Engineering and Applied Chemistry, University of Toronto, Toronto, ON M5S 1A4, Canada; Centre for Environmental Biotechnology, School of Natural Sciences, Bangor University, Bangor LL57 2UW, UK; Department of Biochemistry, University of Toronto, Toronto, ON M5G 1M1, Canada; Department of Molecular Genetics, University of Toronto, Toronto, ON M5G 1M1, Canada; Department of Molecular Genetics, University of Toronto, Toronto, ON M5G 1M1, Canada; Department of Chemical Engineering and Applied Chemistry, University of Toronto, Toronto, ON M5S 1A4, Canada; Department of Chemical Engineering and Applied Chemistry, University of Toronto, Toronto, ON M5S 1A4, Canada; Department of Microbiology, Immunology and Infectious Diseases, Health Research Innovation Centre, University of Calgary, Calgary, AB T2N 4N1, Canada; Department of Molecular Biology and Microbiology, Tufts University School of Medicine, Boston, MA 02115, USA; Department of Chemical Engineering and Applied Chemistry, University of Toronto, Toronto, ON M5S 1A4, Canada; Centre for Environmental Biotechnology, School of Natural Sciences, Bangor University, Bangor LL57 2UW, UK; Department of Biochemistry, University of Toronto, Toronto, ON M5G 1M1, Canada; Department of Molecular Genetics, University of Toronto, Toronto, ON M5G 1M1, Canada

**Keywords:** *Saccharomyces cerevisiae*, random mutagenesis screen, missense mutation, loss-of-function mutant, bacterial effector, *Legionella pneumophila*, AlphaFold

## Abstract

To remodel their hosts and escape immune defenses, many pathogens rely on large arsenals of proteins (effectors) that are delivered to the host cell using dedicated translocation machinery. Effectors hold significant insight into the biology of both the pathogens that encode them and the host pathways that they manipulate. One of the most powerful systems biology tools for studying effectors is the model organism, *Saccharomyces cerevisiae*. For many pathogens, the heterologous expression of effectors in yeast is growth inhibitory at a frequency much higher than housekeeping genes, an observation ascribed to targeting conserved eukaryotic proteins. Abrogation of yeast growth inhibition has been used to identify bacterial suppressors of effector activity, host targets, and functional residues and domains within effector proteins. We present here a yeast-based method for enriching for informative, in-frame, missense mutations in a pool of random effector mutants. We benchmark this approach against three effectors from *Legionella pneumophila*, an intracellular bacterial pathogen that injects a staggering >330 effectors into the host cell. For each protein, we show how in silico protein modeling (AlphaFold2) and missense-directed mutagenesis can be combined to reveal important structural features within effectors. We identify known active site residues within the metalloprotease RavK, the putative active site in SdbB, and previously unidentified functional motifs within the C-terminal domain of SdbA. We show that this domain has structural similarity with glycosyltransferases and exhibits in vitro activity consistent with this predicted function.

## Introduction

For many bacterial pathogens, host manipulation derives from the collective activity of large numbers of translocated proteins (effectors) that are injected into the host cell using dedicated secretion machinery. A striking example is the gram-negative bacterium *Legionella pneumophila* which naturally replicates in freshwater protozoa and is the causative agent of Legionnaires’ disease in humans (Fields *et al*. 2002). The *L. pneumophila* genome encodes the largest effector arsenal described to date (>330 effectors per isolate, or roughly 10% of the proteome) (Burstein *et al*. 2009; Huang *et al*. 2011; Zhu *et al*. 2011), which is injected into the host cell using the Dot/Icm-type IVB secretion system (Segal *et al*. 1998; Vogel *et al*. 1998). *L. pneumophila* effectors modulate very conserved host processes, such as vesicle trafficking, post-translational modification, protein translation, autophagy, vacuolar function, and the cytoskeleton to avoid lysosomal fusion and to establish a replicative, neutral pH vacuole (Isberg *et al*. 2009; Escoll *et al*. 2013; Sherwood and Roy 2016; Qiu and Luo 2017; Mondino *et al*. 2020; Luo *et al*. 2021; Shames 2023; Yang *et al*. 2023). While over 50 effectors have been studied, most of the effectors remain uncharacterized (Finsel and Hilbi 2015; Mondino *et al*. 2020).

Determining the function of the >330 effectors and the role they play in establishing the *Legionella*-containing vacuole is complicated by extensive genetic redundancy within the effector arsenal (O’Connor *et al*. 2011) and the lack of predicted conserved domains or functions for many substrates (Gomez-Valero *et al*. 2011; Gomez-Valero *et al*. 2014; Burstein *et al*. 2016). Only half of the predicted effectors contain conserved domains, and many of these are of uncharacterized function (Burstein *et al*. 2016). Although the amino acid sequence of many effectors may not yield obvious clues to their function, some effectors have structural homology to characterized proteins or domains, along with conserved active site motifs or other signature motifs (Toulabi *et al*. 2013; Morar *et al*. 2015; Wong *et al*. 2015; Urbanus *et al*. 2016; Pinotsis and Waksman 2017; Kozlov *et al*. 2018; Lin *et al*. 2018; Valleau *et al*. 2018; Black *et al*. 2019; Sulpizio *et al*. 2019; Hsieh *et al*. 2021; Voth *et al*. 2021). Looking beyond *L. pneumophila*, over 18,000 effector genes (including orthologs, paralogs, and unique genes) have been predicted across the entire *Legionella* genus (Burstein *et al*. 2016; Gomez-Valero *et al*. 2019). A wealth of novel effector activities and host biology remains to be discovered.

We set out to develop a method to efficiently identify important motifs or amino acid residues in uncharacterized *L. pneumophila* effectors by random mutagenesis and selection for loss-of-function mutations to facilitate the prediction of mechanism and function. As has been observed for the effectors of other pathogens (Lesser and Miller 2001; Valdivia 2004; Siggers and Lesser 2008), the heterologous expression of *L. pneumophila* effectors often leads to inhibition of yeast growth. While the level of inhibition varies between effectors, approximately 10% of the effectors severely inhibit yeast growth when overexpressed (Campodonico *et al*. 2005; Shohdy *et al*. 2005; de Felipe *et al*. 2008; Heidtman *et al*. 2009; Shen *et al*. 2009; Guo *et al*. 2014; Urbanus *et al*. 2016) such that loss-of-function by random mutagenesis can be selected for as an alleviation of the yeast growth defect. However, a random mutant pool contains many mutations that can potentially cause a loss-of-function phenotype, such as frameshift, nonsense, and missense mutations in the effector or regulatory elements such as the promoter region. While most frameshift and nonsense mutations are so disruptive as to be largely uninformative, missense loss-of-function mutations can be extremely informative by identifying specific residues and motifs essential for protein function. To enrich for full-length missense clones, we used a C-terminal in-frame fusion of the yeast *HIS3* gene to effector genes to complement a yeast strain carrying the *his3Δ1* allele (Brachmann *et al*. 1998) and selected for the ability to grow on medium lacking histidine, which requires the presence of a full-length fusion protein. A similar strategy (C-terminal *HIS3* fusions) was previously shown to enrich for in-frame human open reading frames among a randomly primed pool of cDNAs cloned into a yeast expression vector (Holz *et al*. 2001). Here, we benchmark this in-frame mutagenesis approach against three *L. pneumophila* effectors previously shown to inhibit yeast growth (Heidtman *et al*. 2009): SdbA and SdbB, whose functions remain uncharacterized and RavK, a previously described metalloprotease (Liu *et al*. 2017). We show our approach identifies active site residues within RavK (Liu *et al*. 2017), the putative active site in SdbB and previously unidentified functional motifs in the C-terminal domain of SdbA. These motifs are part of the donor- and acceptor-binding regions of glycosyltransferases, which we show share homology with the C-terminal domain of SdbA. Finally, we show that a C-terminal fragment of SdbA exhibits in vitro activity consistent with this predicted function.

## Materials and methods

### In-frame *effector-HIS3* fusion by yeast recombinational cloning

The *Saccharomyces cerevisiae* BY4742 (*MATa, his3Δ1, leu2Δ0, met15Δ0, ura3Δ0*) (Brachmann *et al*. 1998) strains overexpressing *lpg0275*, *lpg0969*, and *lpg2482* (*sdbA*, *ravK*, and *sdbB*, respectively) from the high-copy vector pYES2 NT/A (Life Technologies, GAL 1 promoter, N-terminal 6X HIS/Xpress tag, and URA3 selectable marker) (Heidtman *et al*. 2009) were used to create the *effector-HIS3* fusion mutants by yeast recombinational cloning. The *S. cerevisiae HIS3* gene was PCR amplified from pAG423GAL-ccdB (Alberti *et al*. 2007) using an effector-specific forward primer containing the last 50–60 nucleotides of the effector (minus the stop codon) followed by the first 20–30 nucleotides of the *HIS3* sequence and the pYES-HIS3 reverse primer (Supplementary Table 1). The resulting PCR products were transformed together with XbaI-/PmeI-digested pYES2 NT/A vector encoding *sdbA*, r*avK*, or *sdbB* to BY4742 using the high-efficiency lithium acetate/single-stranded carrier DNA/PEG method (Gietz and Schiestl 2007) and plated onto SD-uracil with 2% glucose (SD-Ura/gluc). The resulting transformants were screened by PCR and sequence verified. To confirm that the *HIS3* fusion does not interfere with effector function, the ability of the effector-His3 fusion protein to cause a yeast growth defect was tested by comparing the growth BY4742 with empty vector control, the wild-type effector, and the effector-His3 fusion in a yeast spot dilution assay as described previously (Urbanus *et al*. 2016).

### Selection of loss-of-function mutations

The *effector-HIS3* fusion vectors were mutagenized in XL-1 Red (Agilent) as per manufacturer's instructions. XL-1 Red transformants were washed off the transformation plate, grown overnight in 50 ml of LB with ampicillin, and the resulting mutant plasmid pool was purified using PureYield Plasmid Midipreps (Promega). The mutant plasmid pool was transformed to BY4742 using the high-efficiency lithium acetate/single-stranded carrier DNA/PEG method (Gietz and Schiestl 2007). Four transformation reactions were performed per screen, each using 1 μg of plasmid pool per reaction. One reaction was split into three parts and plated onto different media types to quantify the transformable (intact selection marker and origin of replication) plasmids (SD-Ura/gluc), loss-of-function mutants [SD-uracil +2% galactose (SD-Ura/gal)], and missense loss-of-function mutants [SD-uracil/histidine + 2% galactose (SD-Ura/His/gal)] and incubated for 2–4 days at 30°C. The three remaining transformation reactions were plated onto 150 mm SD-Ura/His/gal plates and allowed to grow until colonies appeared (3–4 days). Plasmids were rescued from missense loss-of-function mutants and transformed to the *E. coli* Top10 strain before sequencing using primers in the vector and effector, if required (Supplementary Table 1). Details of the sequenced mutants are shown in Supplementary Table 2.

### Analysis of mutant fitness

Liquid growth assays were used to assess the effect of missense loss-of-function mutations on yeast fitness as described (Urbanus *et al*. 2016) with the following modifications. Overnight cultures of freshly transformed BY4742 with empty vector control, pYES2 NT/A *effector-HIS3* wild type and mutants were diluted 100-fold into 100 μl of SD-Ura/gal and grown with Breathe-Easy adhesive seals (EK Scientific) in a CellGrower robot (S&P Robotics) at 30°C with intermittent shaking. Yeast growth was monitored for 30 h by measuring the OD_620_ every 15 min. Growth fitness was calculated as the ratio of the area under the curve (AUC) of an effector-expressing strain over an empty vector control after 30 h using the R package GrowthCurver (Sprouffske and Wagner 2016). The average AUC ratio and standard deviation were calculated from three technical replicates.

### Expression of missense loss-of-function mutants

Expression levels of the missense loss-of-function mutants were assessed by western blot. BY4742 strains with empty vector controls, wild-type *effector*, *effector-HIS3* fusion, and *effector-HIS3* mutant clones were grown overnight in SD-Ura/gluc. To induce expression, 10 OD_600_ units were washed with SD-Ura/gal, resuspended in 5 ml SD-Ura/gal, and grown for 6 h at 30°C. Three OD_600_ units were harvested, treated as described (Zhang *et al*. 2011), resuspended in 100 μl 2X sample buffer, and incubated for 5 min at 95°C. Samples were analyzed using SDS–PAGE and western blot using the following antibodies: anti-Xpress (1:5000, catalog no. R910-25, Invitrogen), anti-actin (1:2500, catalog no. A2066, Sigma-Aldrich), and secondary antibodies anti-mouse HRP (1:5000) or anti-rabbit HRP (1:5000) (Cell Signaling Technology, catalog nos. 7074 and 7076).

### HHpred analysis and sequence alignments

The amino acid sequences of RavK and SdbA (amino acid residues 528–1116) were submitted to the HHpred server (https://toolkit.tuebingen.mpg.de/#/) (Zimmermann *et al*. 2018) analyzed using MSA generation HHblits Uniclust20_2017_07 and Uniprot20_2016_02, respectively, and otherwise default parameters. The resulting alignments were visualized using Boxshade (https://github.com/mdbaron42/pyBoxshade). The HHpred hits on the RCSB Protein Data Bank (https://www.rcsb.org/) (Berman *et al.* 2000) are the following: 4JIU, 4JIX (López-Pelegrín *et al.* 2013) 4QHJ (López-Pelegrín *et al.* 2014), 2L0R (Dalkas *et al.* 2010), 3C37 (Kuzin *et al*. 2008) and 1F0K (Ha *et al*. 2000). Amino acid sequence alignments of SdbB with its orthologs or with SidB were generated using T-coffee (Di Tommaso *et al*. 2011) and visualized using Jalview (Waterhouse *et al*. 2009) or Boxshade.

### Nucleotide sugar donor specificity of the SdbA C-terminal domain

The gene fragment corresponding to SdbA residues 510–1050 was PCR amplified from *L. pneumophila* str Philadelphia-1 genomic DNA and inserted into the pMCSG53 plasmid (Eschenfeldt *et al*. 2013) by ligation-independent cloning, providing an N-terminal 6xHIS-TEV tag. The point mutant E963A was prepared by site-directed mutagenesis using QuikChange site-directed mutagenesis kit (Stratagene) according to the manufacturer's protocol. Plasmids were sequenced and transformed into the *E. coli* BL21 DE3 Gold strain for purification. Recombinant proteins were purified to near homogeneity (>95%) using Ni-chelate affinity chromatography on Ni-NTA Superflow resin (Qiagen) using standard protocols. Cultures were grown in TB, and expression was induced at an OD_595_ of 0.8 with 0.4 mM IPTG overnight at 16°C. Cells were harvested by centrifugation at 9300× *g*, resuspended in 50 mM HEPES pH 7.5, 400 mM NaCl, 5% glycerol, 5 mM imidazole, and lysed by sonication. Lysates were clarified by centrifugation at 21,000× *g* at 4°C and loaded onto gravity flow Ni-NTA agarose columns (Qiagen), followed by washing with 50 mM HEPES pH 7.5, 400 mM NaCl, 5% glycerol, and 30 mM imidazole. Proteins were eluted using 50 mM HEPES pH 7.5, 400 mM NaCl, 5% glycerol, and 250 mM imidazole and flash-frozen in liquid nitrogen for storage at −80°C. The purity of the protein samples was assessed by SDS–PAGE and visualized by Coomassie Brilliant Blue R.

The nucleotide sugar donor specificity of SdbA_510–1050_ was assayed using the UDP-Glo glycosyltransferase assay (Promega) according to the manufacturer's protocol. Briefly, 0.09 µM of purified wild-type and E963A mutant SdbA_510–1050_ protein was incubated with 100 µM UDP-glucose, UDP-GlcNAc, UDP-glucuron, UDP-galactose, or UDP-GalNAc for 1 h at 30°C in 50 mM HEPES, pH 7.5, 100 mM KCl, 2 mM MgCl_2_, and 1 mM MnCl_2_. The hydrolysis of the UDP-substrate was detected as the release of UDP by the UDP-Glo assay (Promega) after 20 min of incubation with UDP-Glo detection reagent. Luminescence was measured using a SpectraMax M2 plate reader. Three technical repeats were performed per reaction.

The *V*_max_, *K_m_*, and *k*_cat_ for wild-type SdbA_510–1050_ with UDP-GlcNAc was determined by incubating 0.16 µM SdbA_510–1050_ with UDP-GlcNAc concentration range of 0.0039–2 mM for 1 h at 30°C in 50 mM HEPES, pH 7.5, 100 mM KCl, 2 mM MgCl_2_, and 1 mM MnCl_2_. Three technical repeats were performed per reaction. Kinetic parameters were determined by non-linear curve fitting from the Michaelis–Menten plot using GraphPad Prism (version 5.00 for Windows, GraphPad Software).

## Results

### A random mutagenesis screen to identify regions important for bacterial effector function

To efficiently screen for randomly generated mutations in *L. pneumophila* effectors that cause a loss-of-function phenotype and represent full-length protein rather than frameshift or nonsense mutations, we applied a yeast method first designed to select for human cDNA inserts that contain intact open reading frames (Holz *et al*. 2001). This method leverages tools developed for plasmid selection and protein expression in the model organism *S. cerevisiae* (budding yeast). A strain with several auxotrophic alleles (*his3Δ1, leu2Δ0, met15Δ0, ura3Δ0;* genes involved in histidine, leucine, methionine, and uracil pathways) (Brachmann *et al*. 1998) allows for maintenance of a yeast plasmid encoding the wild-type allele as a selection marker. The wild-type alleles complement the auxotrophic allele and allow growth on medium lacking histidine, leucine, methionine, or uracil (Sikorski and Hieter 1989).

**Fig. 1.**
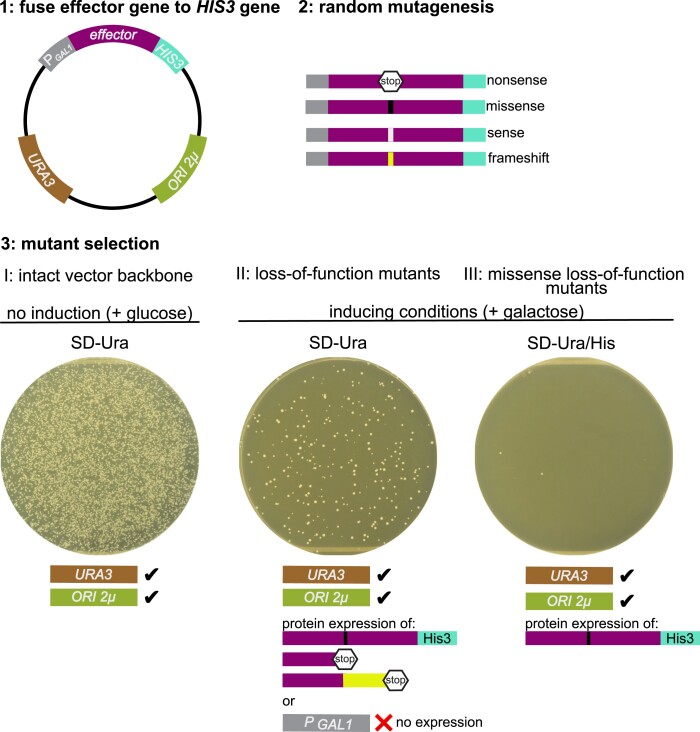
A method to enrich full-length missense mutants in a random mutagenesis screen. A schematic representation of the random mutagenesis screen enriched for missense mutations. 1: *L. pn*e*umophila* effector genes causing a severe growth phenotype when expressed in yeast were fused in-frame to the *S. cerevisiae HIS3* gene on a high-copy (ORI 2µ) yeast expression vector with galactose-inducible expression (P*_GAL1_*) and an uracil selection marker (*URA3*). 2: The plasmids were randomly mutated in the *E. coli* mutator strain XL-1 Red. The resulting mutant pool contained a variety of mutations: sense, nonsense, missense, and frameshifts (yellow line), of which the latter three can cause loss-of-function phenotypes. 3: The plasmid pool was transformed to the *S. cerevisiae* strain BY4742 and grown under conditions that selected for an intact vector backbone (I, SD-Ura with glucose), all loss-of-function mutations (II, SD-Ura with galactose induction), and for expression of full-length effector-His3 fusion proteins caused by missense loss-of-function mutations (III, SD-Ura/His with galactose induction). The 100-mm plate images each represent 8% of the transformation pool used in the *sdbB* screen.

We cloned the *S. cerevisiae HIS3* gene in-frame behind *L. pneumophila* effector genes on a high-copy galactose-inducible yeast expression plasmid with a *URA3* selection marker (Fig. 1). After confirming that the C-terminal His3 fusion does not interfere with the yeast growth phenotype and therefore likely does not interfere with effector function, we generated a pool of random mutants using the *E. coli* mutator strain XL-1 Red. We then transformed this mutant plasmid pool to yeast and monitored growth on different media types. To assess the number of vectors with an intact backbone, where the *URA3* marker can complement the *ura3Δ0* allele of BY4742 and the vector has an intact origin of replication (ORI 2µ), the transformed pool was grown on medium lacking uracil and with glucose (SD-Ura/gluc) to repress expression of the *effector-HIS3* fusion (Fig. 1, step 3I). To look at the efficiency of the random mutagenesis step, we grew the transformed pool on medium lacking uracil and with galactose to induce expression of the *effector-HIS3* fusion (SD-Ura/gal) selecting all mutations that caused a loss of function in the effector (Fig. 1, step 3II). This can be caused by promoter mutations that disrupt expression or missense, nonsense, or frameshift mutations. Finally, to specifically select for full-length missense loss-of-function mutation, we grew the transformed pool on medium with galactose and lacking uracil and histidine (SD-Ura/His/gal), which requires the production of full-length effector-His3 fusion protein and mutations in the effector gene that disrupt effector activity (Fig. 1, step 3III).

### Missense loss-of-function screen identifies important conserved SdbB residues

**Fig. 2.**
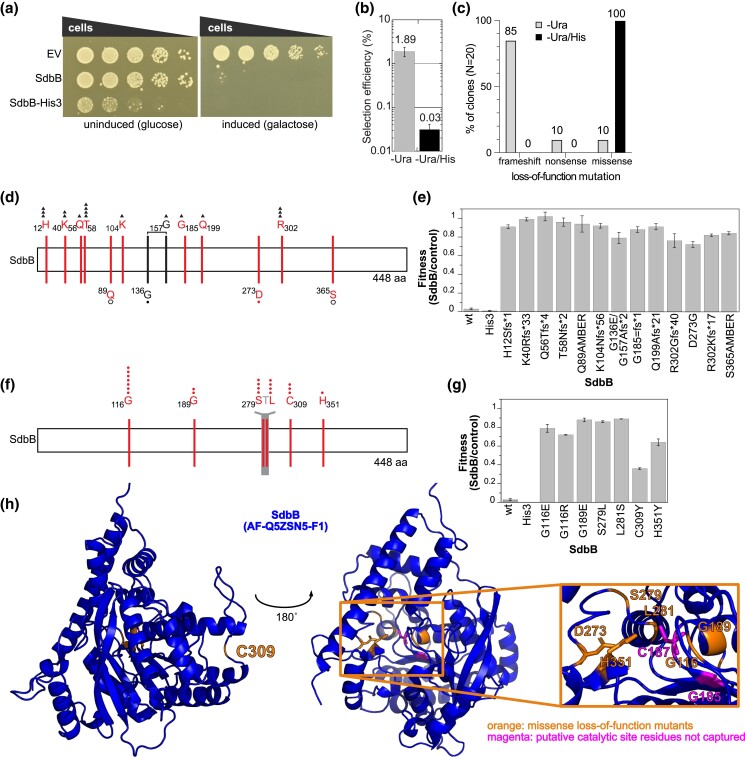
The full-length ORF-enriched random mutagenesis screen identifies missense mutations in the putative active site of SdbB. a) Expressing *sdbB* and the *sdbB* in-frame fusion with *HIS3* caused a severe yeast growth defect as shown in a yeast spot dilution assay. A dilution series of yeast strains carrying an empty vector or plasmids with *sdbB* and *sdbB-HIS3* were spotted on SD-Ura/gluc (uninduced condition) and SD-Ura/gal (inducing conditions) and grown 2 days at 30°C before imaging. b) The percentage of loss-of-function mutations in the pool of transformable plasmids selected on SD-Ura/gal (gray) and SD-Ura/His/gal (black) medium. The average and standard deviation of three independent replicates are shown. c) Percentage of the occurrence of frameshift, nonsense, and missense mutations in 20 sequenced clones selected on SD-Ura/gal (gray) and SD-Ura/His/gal (black). d) A schematic representation of loss-of-function clones selected on SD-Ura/gal. Mutations recovered from the same clone are shown in black and are connected by a horizontal line, while single mutations are shown in red. Mutation type is indicated as a closed triangle (frameshift), open hexagon (nonsense), or closed circle (missense), and the number of symbols reflects the occurrence of the mutation in the dataset. e) The fitness of wild-type *sdbB*, *sdbB-HIS3* fusion, and loss-of-function mutant strains (selected on SD-Ura/gal) compared to empty vector controls confirmed the loss-of-function phenotype for the *sdbB* random mutagenesis clones. The fitness was determined using growth curve assays (see methods) and calculated as the ratio (wt or mt *sdbB*/empty vector control) of the area under the growth curve at 30 h. The average and standard deviation of three technical replicates are shown. f) A schematic representation of SdbB loss-of-function clones selected on SD-Ura/His/gal. Missense loss-of-function mutations are shown in red with a closed circle; the number of symbols reflects the occurrence of the mutation in the dataset. Amino acids shown for presentation purposes are shown in gray. g) The fitness of wild-type *sdbB*, *sdbB-HIS3* fusion and loss-of-function mutants (selected on SD-Ura–His/gal) compared to empty vector controls in liquid growth assays confirms the loss-of-function phenotype for the *sdbB* random mutagenesis strains. The average and standard deviation of three technical replicates are shown. h) The missense mutations identified by the random mutagenesis screen (d, f) are shown in orange on an Alphafold2 model of SdbB (AF-Q5ZSN5-F1-model_v4.pdb), and residues from the putative active site G185xCxG189 not captured by the screen are shown in magenta. Putative catalytic triad C187-D273-H351 residues are shown with sticks and the box shows the enlargement of the putative catalytic site. The AlphaFold2 model was visualized using the PyMOL Molecular Graphics System, version 2.2, Schrödinger, LLC.

As a proof of principle, we looked at *sdbB* which causes a severe yeast growth phenotype when expressed (Fig. 2a) (Heidtman *et al*. 2009) and is part of the *sidB* paralog family, whose members are predicted to be lipases from the α/β hydrolase enzyme family (Luo and Isberg 2004). After verifying that the *sdbB-HIS3* fusion was still capable of causing a yeast growth defect (Fig. 2a), we created an *sdbB-HIS3* random mutagenesis pool and quantified the number of CFUs on the different selection media. While 1.89% of the transformable plasmids carried a mutation that allowed for growth on SD-Ura/gal medium indicating some type of loss-of-function mutation (Fig. 2b), only 0.03% of the transformable plasmids carried a mutation allowing growth on SD-Ura/His/gal medium—a condition that requires the expression of a full-length fusion protein. The efficiency of the histidine selection step was verified by sequencing 20 clones from each condition. The loss-of-function clones selected on SD-Ura/gal consisted of 16 frameshift mutations, 2 nonsense mutations, 1 combination of a missense and frameshift mutation, and 1 missense mutation in the *sdbB* gene (Fig. 2c and d). In contrast, all 20 loss-of-function clones selected on SD-Ura/His/gal contained only missense mutations (Fig. 2c and f). In both conditions, a number of mutations were recovered several times, suggesting that sequencing additional clones would yield few new mutations. To confirm that the identified mutations indeed rescued the *sdbB*-induced growth defect in yeast, we compared the growth of *sdbB* wild type and mutants to an empty vector control in a liquid growth curve assay. Using the area under the growth curve (AUC) at 30 h, which encompasses differences in every growth phase, we calculated the fitness of the *sdbB* strains as the ratio AUC of *sdbB*/AUC empty vector control. The wild-type and *HIS3*-fused *sdbB* caused a severe yeast growth defect, while the *sdbB* loss-of-function mutants showed a fitness of 60–90% compared to the empty vector control (Fig. 2e and g).

The positions of the frameshift and nonsense mutations in SdbB (Fig. 2d) indicate that a large part of the protein is required for function, as even a nonsense mutation at S365, 84 amino acid residues from the C-terminus, almost completely rescued activity. The missense loss-of-function mutations (Fig. 2d and f) target three amino acid residues (SD-Ura/gal: D273 and SD-Ura/His/gal: G116, H351) that are invariant in SdbB orthologs from *L. pneumophila* and other *Legionella* species (Burstein *et al*. 2016) (Supplementary Fig. 1), suggesting they are essential for function or structure. The G189E mutation is part of the GXS/CXG motif that is highly conserved across the SdbB orthologs and is predicted by NCBI Conserved Domain search (Marchler-Bauer *et al*. 2017) to align with the so-called nucleophile elbow of the nucleophile–acid–base triad of the α/β hydrolase active site (Brenner 1988; Ollis *et al*. 1992; Schrag and Cygler 1997) (Supplementary Fig. 2).

When the missense mutants are mapped onto the SdbB AlphaFold2 model (Jumper *et al*. 2021; Varadi *et al*. 2021), all but C309 localize in the vicinity of the putative catalytic cysteine (C187), including the invariant D273 and H351 residues captured in the screen, suggesting they are the remaining residues of the catalytic triad (Fig. 2h). A key strength of forward genetic approaches is to identify functionally important residues independent of bioinformatics predictions and conservation. Some of the functionally important residues that we identify using our method are conserved and some are not. As an example of the latter, two loss-of-function mutations targeting residues S279 and L281 localize near conserved residues within the SdbB AlphFold2 model (consistent with their apparently essential role in protein function) yet are themselves not conserved between *sdbB* orthologs. We verified expression of all missense loss-of-function mutants, and all are expressed at a higher level than wild-type *sdbB* with C309 having the lowest expression (Supplementary Fig. 3a).

Thus, the *sdbB* example demonstrates that functionally important amino acid residues can be efficiently identified using the random mutagenesis method in conjunction with the histidine selection for full-length protein. Importantly, this approach significantly reduces the number of sequenced clones required to identify amino acid residues or regions of interest, by approximately 60-fold in the case of *sdbB.*

### Missense loss-of-function screen identifies the active site of the characterized effector RavK

**Fig. 3.**
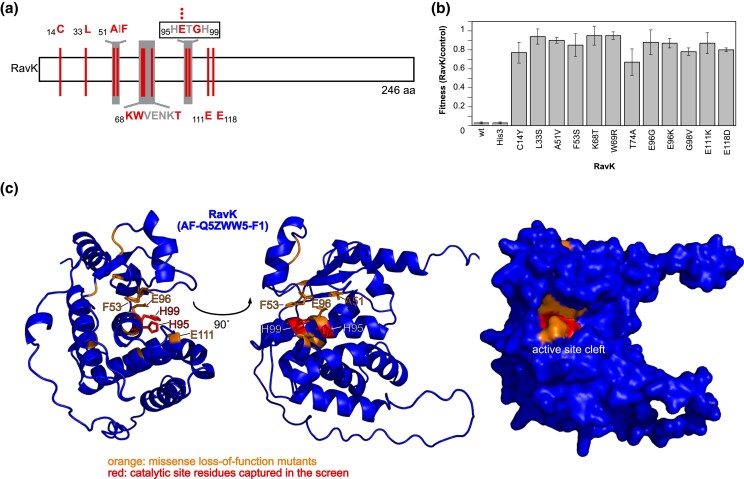
*ravK* random mutagenesis captures residues lining the RavK active site cleft. a) A schematic representation of RavK and the amino acids changed by mutations causing a *ravK* loss-of-function phenotype when expressed in yeast. Mutated residues are shown in red, amino acids shown for presentation purposes are in gray, and the number of symbols reflects the occurrence of the mutation in the dataset. b) The fitness of wild-type *ravK*, *ravK-HIS3* and loss-of-function mutants confirms the loss-of-function phenotype for the *ravK* random mutagenesis strains. The fitness was calculated as the ratio (wt or mt *ravK*/empty vector control) of the area under the growth curve at 30 h. The average and standard deviation of three technical replicates are shown. c) The Alphafold2 model of RavK (AF-Q5ZWW5-F1-model_v4.pdb) with missense mutations shown in orange. The histidine residues of the active site motive H95-Exx-H99 (Liu *et al*. 2017) not captured by the screen are shown in red. The residues in the active site cleft are shown as sticks. The AlphaFold2 model was visualized using the PyMOL Molecular Graphics System, version 2.2, Schrödinger, LLC.

To benchmark the missense loss-of-function screen on a more characterized effector, we looked at *ravK* which also causes a severe yeast growth defect (Heidtman *et al*. 2009; Liu *et al*. 2017). RavK is a small, soluble metalloprotease that specifically cleaves host actin, and directed substitutions within the predicted active site motif HExxH abolish both its activity and toxicity to yeast (Liu *et al*. 2017). After confirming that the *ravK-HIS3* fusion was still able to cause a yeast growth when expressed (Fig. 3b), we subjected this plasmid to random mutagenesis, transformed the pool into yeast, and then selected for loss-of-function mutants on SD-Ura/His/gal medium. Of the 14 loss-of-function clones we sequenced, one clone contained a large, in-frame deletion from amino acid residue 70 to residue 166, which was unexpected but confirms the strength of the histidine selection for maintaining open reading frames. All other loss-of-function clones were caused by single point mutations resulting in missense mutations (Fig. 3a). In the growth assay, wild-type and *HIS3*-fused *ravK* almost completely inhibited yeast growth, while the *ravK* mutants displayed a fitness of 70–90% compared to the empty vector control. All *ravK* mutants are expressed at a similar level (Supplementary Fig. 3b). The loss-of-function mutations all map to the first half of RavK, suggesting that the N-terminal half of RavK is essential for RavK function. This agrees with the previous study which identified the active site motif (H95ExxH99) in the N-terminal half of the protein and found that the 50 C-terminal residues of RavK can be deleted without any effect on its activity on actin (Liu *et al*. 2017).

Four of the loss-of-function mutations that we isolated targeted the active site motif H95ExxH99, three of which are mutations in the E96 codon. Our screen also identified several residues outside of this motif that are critical for RavK function. To investigate why these might be functionally important, we performed an HHpred analysis which looks for structural homologs of proteins (Zimmermann *et al*. 2018). HHpred identified many hits with homology to the HExxH metalloprotease motif. Among the top five HHpred hits are three small soluble metalloproteases or minigluzincins, anthrax lethal factor, and a zinc-dependent peptidase from the M48 family (Kuzin *et al.* 2008; Dalkas *et al*. 2010; López-Pelegrín *et al*. 2013; López-Pelegrín *et al*. 2014) (Supplementary Fig. 4). Notably, some of the other loss-of-function mutations occur in areas that have homology with structural elements in the minigluzincins contributing to the active site cleft (López-Pelegrín *et al*. 2013) (Supplementary Fig. 4). Indeed, when the missense mutations are mapped on the RavK AlphaFold2 model (Jumper *et al*. 2021; Varadi *et al*. 2021) (Fig. 3c), they are localized around the active site HExxH including the top rim of the active site cleft.

### The C-terminal domain of SdbA is a putative glycosyltransferase

Like *sdbB*, *sdbA* is a member of the s*idB* paralog family (Luo and Isberg 2004). While the function of SdbA remains undefined, experimental evolution of *Legionella* in mouse macrophages selected for parallel *sdbA* nonsense and frameshift mutations in three out of four independent lineages (Ensminger *et al*. 2012), suggesting that SdbA activity partially restricts growth in this accidental host. While the N-terminal domain of SdbA has homology with SidB (Luo and Isberg 2004), the additional C-terminal domain does not have significant sequence homology to other known proteins (data not shown). Expression of *sdbA* completely inhibits yeast growth (Heidtman *et al*. 2009), making the missense loss-of-function screen an informative tool to identify functional residues that might suggest a specific activity inside the eukaryotic cell.

**Fig. 4.**
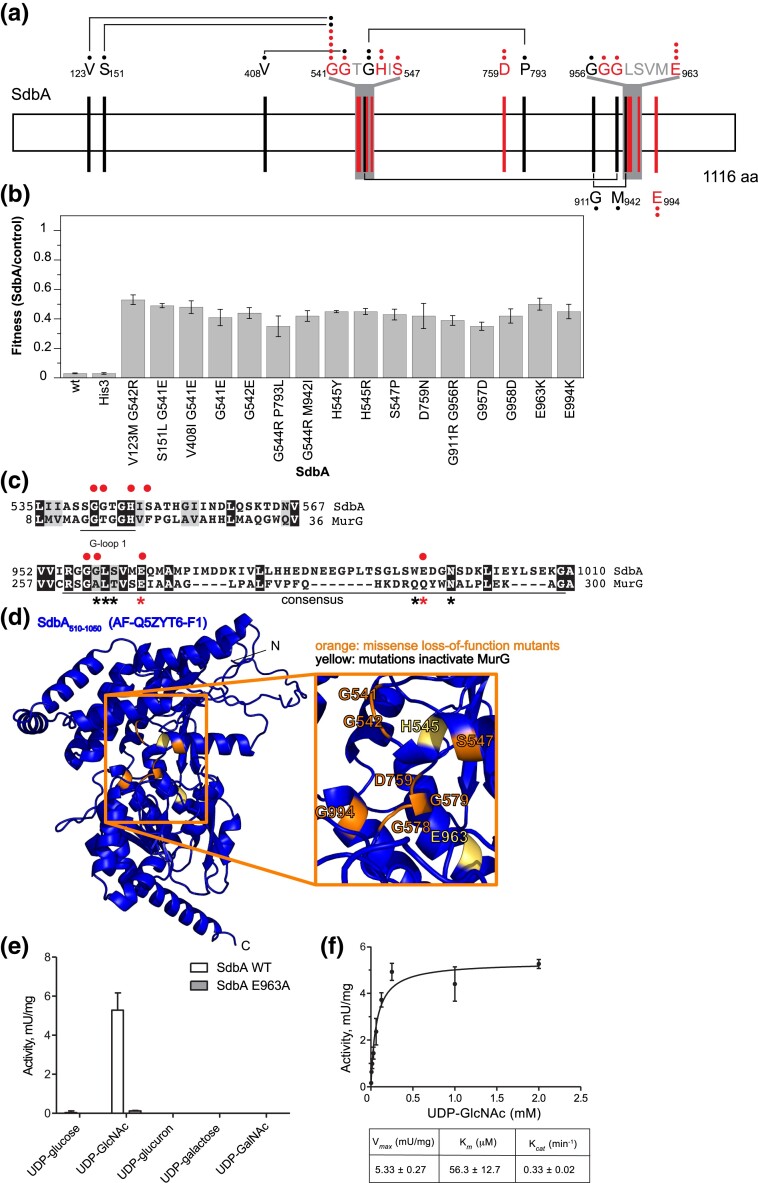
The C-terminal domain of SdbA is a putative glycosyltransferase domain. a) A schematic representation of SdbA and the residues changed by the mutations causing a s*dbA* loss-of-function phenotype when expressed in yeast. Mutations recovered from the same clone are shown in black, connected by a line, and single mutations alleviating an *sdbA*-induced growth defect are shown in red. Amino acids shown for presentation purposes are shown in gray. Black and red closed circles indicate the number of times the mutation was identified. b) The fitness of wild-type *sdbA*, *sdbA-HIS3*, and loss-of-function mutants compared to empty vector controls. The fitness was calculated as the ratio (wt or mt *sdbA*/empty vector control) of the area under the growth curve at 30 h. *sdbA* and *sdbA-HIS3* display a severe growth defect, while the loss-of-function mutations rescue growth up to 50% of the empty vector control. The average and standard deviation of three technical replicates are shown. c) HHpred alignment of SdbA with PDB:1F0K, *E. coli* MurG (Ha *et al*. 2000). The G-loop 1 and consensus sequence are shown with identical residues (black) and similar residues (gray) highlighted. SdbA residues that when mutated abrogate activity are indicated by a red closed circle. Residues in the MurG consensus sequence that contact UDP-GlcNac are indicated by a black star, or a red star if mutating the residue abrogates MurG activity. d) The Alphafold2 model of SdbA (AF-Q5ZYT6-F1-model_v4.pdb) with missense mutations shown in orange. Residues H545 and E963 corresponding to residues H18 and E268 in MurG are shown in yellow. The AlphaFold2 model was visualized using the PyMOL Molecular Graphics System, version 2.2, Schrödinger, LLC. e) The SdbA glycosyltransferase domain uses GlcNAc as a donor substrate. Donor substrate specificity was tested by incubating 0.09 µM of purified fragment (residues 510–1050) of wild-type SdbA or an inactive mutant (E963A) with 100 µM UDP-glucose, UDP-GlcNAc, UDP-glucuron, UDP-galactose, or UDP-GalNAc for 1 h at 30°C. The hydrolysis of the UDP-substrate was detected as the release of UDP by the UDP-Glo assay (Promega) after 20 min of incubation with UDP-Glo detection reagent. Luminescence was measured using a SpectraMax M2 plate reader, and the hydrolysis activity was calculated as mU (nmoles UDP-substrate/min) per mg SdbA_510–1050_ wt or E963 K. Three technical repeats were performed per reaction. f) Determination of the kinetic parameters for GlcNAc hydrolysis by SdbA_510–1050_. Reactions with 0.16 µg purified wild-type C-terminal domain of SdbA (residues 510–1050) and a range of 0.0039–2 mM GlcNAc were incubated for 1 h at 30°C. GlcNAc hydrolysis was measured using the UDP-Glo glycosyltransferase assay as described above; three technical replicates were performed per reaction. Kinetic parameters were determined by nonlinear curve fitting from the Michaelis–Menten plot.

The missense loss-of-function screen in *sdbA* identified 19 mutations in 24 sequenced clones targeting 17 codons (Fig. 4a). In contrast to the smaller genes *sdbB* and *ravK*, the *sdbA* results included several double mutants. Some of these mutations were also recovered as a single loss-of-function mutant with a similar fitness (Fig. 4b). The *sdbA* mutant clones are expressed at a higher (albeit varying) level than wild-type *sdbA*, which could not be detected by western blot (Supplementary Fig. 3c). All the single mutations that lead to a loss-of-function phenotype fall in the C-terminal domain and concentrate in two regions: G541-GTGHI-S547 and G957-GLSVM-E963. An HHpred homology search (Zimmermann *et al*. 2018) predicted with high confidence that the C-terminal domain is a glycosyltransferase of the GT-B fold. When comparing the SdbA C-terminal domain with the sequence of *E. coli* MurG, a well-studied member of the GT-B fold glycosyltransferase family, the two mutated regions align with the G-loop 1 and a consensus region in GT-B fold superfamily involved in binding the donor molecule (Ha *et al*. 2000; Hu *et al*. 2003; Crouvoisier *et al*. 2007) (Fig. 4c). Glycosyltransferases hydrolyze UDP-sugar donor molecules and transfer the sugar to the acceptor molecule, which can be a variety of molecules such as small molecules, lipids, or proteins (Lairson *et al*. 2008). In MurG, residues A263, L264, L265, E268, Q287, and Q288 contact the donor molecule UDP-GlcNAc (Hu *et al*. 2003) (Fig. 4c), while the G-loop 1 is thought to be involved in acceptor molecule binding (Ha *et al*. 2000). Mutations in these motifs abrogate MurG enzymatic activity, including mutation of the residues H18 and E268 (Hu *et al*. 2003; Crouvoisier *et al*. 2007), whose corresponding residues in SdbA (H545 and E963) were found to be mutated in our screen. The single missense mutants abrogating SdbA activity were mapped onto the AlphaFold2 model (Jumper *et al*. 2021; Varadi *et al*. 2021) of a SdbA C-terminal fragment (residues 510–1050) with the residues H545 and E963 highlighted in yellow.

To test whether the C-terminal domain of SdbA is indeed a glycosyltransferase, we purified the C-terminal fragment (residues 510-1050) and the equivalent of the MurG E268A inactive mutant in SdbA (E963A) and tested several UDP-sugars as substrate using the UDP-Glo assay (Fig. 4e). Glycosyltransferases can hydrolyze UDP-sugars in the absence of an acceptor molecule (with water acting as an acceptor in the reaction) (Sheikh *et al*. 2017; Vicente *et al*. 2023). Indeed, wild-type SdbA_510–1050_ hydrolyzes the UDP-GlcNAc donor, while the E963A mutant does not (Fig. 4e). This suggests that SdbA is a glycosyltransferase with specificity for UDP-GlcNAc and that a mutation in the E963 codon identified by the missense loss-of-function screen abrogates that activity. Using the same assay, we determined the kinetic parameters of UDP-GlcNAc hydrolysis by SdbA_510–1050_ (Fig. 4f), which revealed high affinity (low micromolar *K_m_*) of the enzyme to UDP-GlcNAc. Taken together, these data support the prediction of SdbA glycosyltransferase activity and demonstrate the power of missense mutations and in silico predictions to inform the functional determination of effector activity.

## Discussion

The identification of functionally important residues through amino acid substitutions is a common tool to interrogate protein activities, test structural predictions, and define protein–protein interaction interfaces. This is typically done through site-directed mutagenesis (reverse genetics) or random mutagenesis (forward genetics). One advantage of random mutagenesis is that it can identify functionally important residues that might be missed by the site-directed mutagenesis approach (e.g. due to lack of apparent sequence homology or incomplete/inaccurate bioinformatics predictions). Nonsense mutations and frameshifts provide limited insight into protein structure or function yet typically make up the majority of loss-of-function mutations recovered after random mutagenesis of a protein sequence. Here, we demonstrate that the combination of a random mutagenesis loss-of-function screen with a selection for full-length protein is highly effective in specifically selecting for loss-of-function missense clones. In fact, all but one of the clones recovered in our assay contained missense mutations, while the remaining one contained an in-frame deletion which included the active site of RavK. The percentage of missense mutant clones for *sdbB* was 0.03% of transformable plasmids, but this number will be different for each gene and experiment. It will depend on the efficiency of the mutagenesis step, the gene length, and the number of functionally important codons that can be mutated by a single mutation. We recommend performing one yeast transformation with a new mutant plasmid pool and plate one-third on the different selection media as described in materials and methods (SD-Ura/gluc, SD-Ura/gal and SD-Ura/His/gal). This will indicate whether the random mutagenesis worked and gauge how many yeast transformations are needed to isolate sufficient loss-of-function clones.

We have benchmarked this approach against three effectors in *L. pneumophila* using existing structural information and advances in protein modeling (AlphaFold) to reveal how a forward genetic approach can both validate existing knowledge and models and reveal novel functional residues that would be difficult to predict using only homology or modeling alone. Notably, our approach correctly identified residues in the previously described active site of RavK, the predicted active site nucleophile motif of SdbB, and the remaining putative residues of the catalytic triad of SdbB. Forward genetics can also lead to unexpected results; in our case, this came in the form of insight into the functional divergence of two apparent paralogs. While our approach enriched for several loss-of-function mutations within the α/β hydrolase domain of *sdbB*, in *sdbA*, the loss-of-function mutations that we recovered localized to its C-terminal domain. Based on this mutational profile, we were able to predict that the C-terminal domain of SdbA is a glycosyltransferase, a hypothesis supported by in vitro activity toward UDP-GlcNAc. For proteins with even less structural information, such mutational profiling can focus follow-up studies designed to link specific domains to activity.

A close examination of our data shows that not all important functional residues were identified in the screens, especially those within the active site residues of RavK and SdbB. This suggests that the mutational space of these proteins has not been saturated, though from a practical perspective we recovered several identical mutations in each, suggesting diminishing returns of sequencing additional clones. To expand sampling of the mutational space, an alternative method of random mutagenesis such as error-prone PCR could be used to increase the number of mutations. In a direct comparison of these methods, error-prone PCR introduced more mutations than the XL-1 Red mutator strain, though the incidence of multiple mutations per clone would increase (Rasila *et al*. 2009).

To effectively apply the random mutagenesis missense enrichment selection or extend this approach to bacteria and mammalian cells, several considerations should be taken into account. First, the protein of interest must have a selectable loss-of-function phenotype such as alleviation of growth defect. Growth fitness is a universal phenotype that is easy to measure in bacteria, yeasts and mammalian cell lines. Second, the C-terminal fusion of a selection marker must not interfere with protein function. If the function of protein of interest is inhibited by the C-terminal fusion, it could potentially be overcome by introducing linker regions of varying length and flexibility (Chen *et al*. 2013) or by using a cleavable linker such as the ubiquitin K0 mutant that is processed by cytosolic deubiquitinases in eukaryotic cells (Bachran *et al*. 2013). Similarly, the C-terminal selection marker must be able to function as a fusion protein or be liberated by an in vivo cleavable linker. To extend the random mutagenesis missense enrichment selection to bacteria, the chloramphenicol acetyltransferase (CAT) gene (which confers resistance to chloramphenicol) is a viable candidate as a C-terminal fusion partner. CAT has been successfully used in protein fusions where it conferred chloramphenicol resistance during colony selection as a C-terminal fusion partner, with increased selection efficiency when mutant fusion proteins were soluble (Maxwell *et al*. 1999). In mammalian cell lines, a positive selection marker such as blasticidin S deamidase could be used as a C-terminal fusion partner. Blasticidin S deamidase is functional as a C-terminally fused protein (Suarez and McElwain 2009) and confers resistance against blasticidin, which rapidly inhibits mammalian cell growth at a low dose (Kimura *et al*. 1994). An alternative, if no positive selection marker is available, is GFP, which has been used extensively as a fused localization marker for various cellular compartments and organisms (Margolin 2000; van Roessel and Brand 2002; Huh *et al*. 2003). After a standard number of generation doublings, GFP-positive cells, indicative of the presence of full-length protein, can be isolated by fluorescence-activated cell sorting.

Our initial results suggest that missense-directed mutagenesis will be a useful tool to help identify potential functions for other bacterial effector proteins, many of which have low sequence homology to characterized proteins (Gomez-Valero *et al*. 2011; Burstein *et al*. 2016). Rather than being replaced by in silico protein modeling, we show how the two methodologies complement one another and can be used to identify structural features or regions essential for activity against the eukaryotic cell. In some cases, the combined information of functional residues, protein models, or sequence conservation may not indicate an apparent activity or function of the effector. Even in these cases, the loss-of-function mutants may prove useful in other assays with growth-based readouts.

In *L. pneumophila* alone, 10% of the translocated effectors cause severe yeast growth defects (Campodonico *et al*. 2005; Shohdy *et al*. 2005; de Felipe *et al*. 2008; Heidtman *et al*. 2009; Shen *et al*. 2009; Guo *et al*. 2014; Urbanus *et al*. 2016) and are possible candidates for the random mutagenesis missense enrichment screen. Additional growth phenotypes are likely to be revealed under other conditions of growth, such as nocodazole, caffeine, high osmolarity, pH, low and high temperature, or yeast deletion strains which are known to potentiate some bacterial effectors (Sisko *et al*. 2006; Slagowski *et al*. 2008; Xu *et al*. 2010; Bosis *et al*. 2011; Nevo *et al*. 2014). Identifying functional residues within uncharacterized effectors is a logical first step toward validating in silico protein models, predicting effector activity, and designing protein–protein interaction studies.

## Supplementary Material

jkae158_Supplementary_Data

## Data Availability

Strains and plasmids are available upon request. Supplementary Figure 1 shows an amino acid alignment of SdbB orthologs from seven *Legionella* species along with the location of missense mutations identified in this screen. Supplementary Figure 2 shows an amino acid alignment of SidB and SdbB. Supplementary Figure 3 contains western blots of effector-HIS3 wild type and loss-of-function mutants. Supplementary Figure 4 shows an HHpred alignment of RavK with metalloproteases. Supplementary Figure 5 shows the purity of the SdbA fragments used in the UDP-Glo Glycosyltransferase assay. Supplementary Table 1 shows the primers used for yeast recombinational cloning and sequencing. Supplementary Table 2 lists all the mutations identified in this study. Supplemental material available at G3 online.
