# Supplementary material for: A random mutagenesis screen enriched for missense mutations in bacterial effector proteins
Source: G3 (Bethesda). 2024 Jul 19;14(9):jkae158. doi: 10.1093/g3journal/jkae158 (PMC11373652; doi:10.1093/g3journal/jkae158)
Supplement: jkae158_Supplementary_Data [file jkae158_supplementary_data.zip › Figure S4 240725.pdf]

```
                                                                      β2
ravK       1 MVSLEHIQKLISECRKLGKDGLDNGTNGLIPELEIDVVPPSAFLG-VGNNPAIFVNSKTY
4QHJ_B     1 MKDRKILNEILSNTINE------LNLNDKKANIKIKIKPLKRKIA-SISL----------
4JIU_A     1 -------------------------------------------LG-YQRPVKVRIRPLK-
4JIX_B     1 -------------------------------------------LN-INESINIEIKPMK-
2L0R_A     1 ------------------------------------------------------------
3C37_A     1 -VNDPEVQRYVDKVGKRLLSGARAV----EFDYVFKVVKDDSVNAFAIPGGRVYVHTGLL
            rim of active site cleft
                           β3
ravK      60 KLMRTTHEKWVENKTIVFKSYLL-S-QPAIKIIGAIVHETGHAFN------------V--
4QHJ_B    44 -----------TNKTIYINKNILPY-LSDEEIRFILAHELLHLKY------------GKY
4JIU_A    16 ---MSIARVSFKYGTITLDPAVL-N-LEEEEMFYILIHELAHLKAETSYHSSSFWREV--
4JIX_B    16 ---QKIASFSFKTKTLRLNKYVV-ENFDEELLHYIILHELIHFKI---------------
2L0R_A     1 ------------------------------NDSEGFIHEFGHAVD------------D--
3C37_A    56 KAAD-----------------------NETELAGVLAHEINHAVA---------------

ravK     104 -----AAKIPNT-EANACIFEIEVLMRLFQVKSPLLLGCTELDMQSYFKSRLTDYNKC--
4QHJ_B    80 HINEFEEELLFL-FPNKEAILFNLINKLFQKK----------------------------
4JIU_A    69 -----EKVFPGE-R--AKEIEDRIMTKLQR------------------------------
4JIX_B       ------------------------------------------------------------
2L0R_A    17 -----YAGYLLDKNQSDLVTNSKKFIDIFKEEGSNLTSYGRTNEAEFFAEAFRLMHSTDH
3C37_A       ------------------------------------------------------------

ravK     156 ------VKDCQCLAEMVEFITHQ
4QHJ_B       -----------------------
4JIU_A       -----------------------
4JIX_B       -----------------------
2L0R_A    72 AERLKVQKNAPKTFQFINDQIKF
3C37_A       -----------------------
```

Figure S4: HHpred alignment of RavK with metalloproteases. Alignment of the HHpred top hits; MJ1213 (PDB:4QHJ) (M. López-Pelegrín et al., Angewandte Chemie Int Ed. 53, 10624–10630, 2014), proabylysin (PDB:4JIU), projannalysin (PDB:4JIX) (M. López-Pelegrín et al., J. Biol. Chem. 288, 21279–21294, 2013), anthrax lethal factor (PDB:2L0R) (G. A. Dalkas et al., Biochemistry. 49, 10767–10769, 2010) and a zinc-dependent endopeptidase of the M84 family (PDB:3C37) (A.P. Kuzin, et al., 2008. RCSB Protein Data Bank. https://doi.org/10.2210/pdb3C37/pdb) with RavK is shown where grey and black background indicate the level of amino acid residue conservation as 50% or more similarity or 50% or more identity, respectively. Structural elements of proabylysin (4JIU) are indicated on top of the alignment and mutations that abolish RavK activity are indicated by a red closed circle.
