# Supplementary material for: A random mutagenesis screen enriched for missense mutations in bacterial effector proteins
Source: G3 (Bethesda). 2024 Jul 19;14(9):jkae158. doi: 10.1093/g3journal/jkae158 (PMC11373652; doi:10.1093/g3journal/jkae158)
Supplement: jkae158_Supplementary_Data [file jkae158_supplementary_data.zip › Figure_S1_G3-2024-405229.pdf]

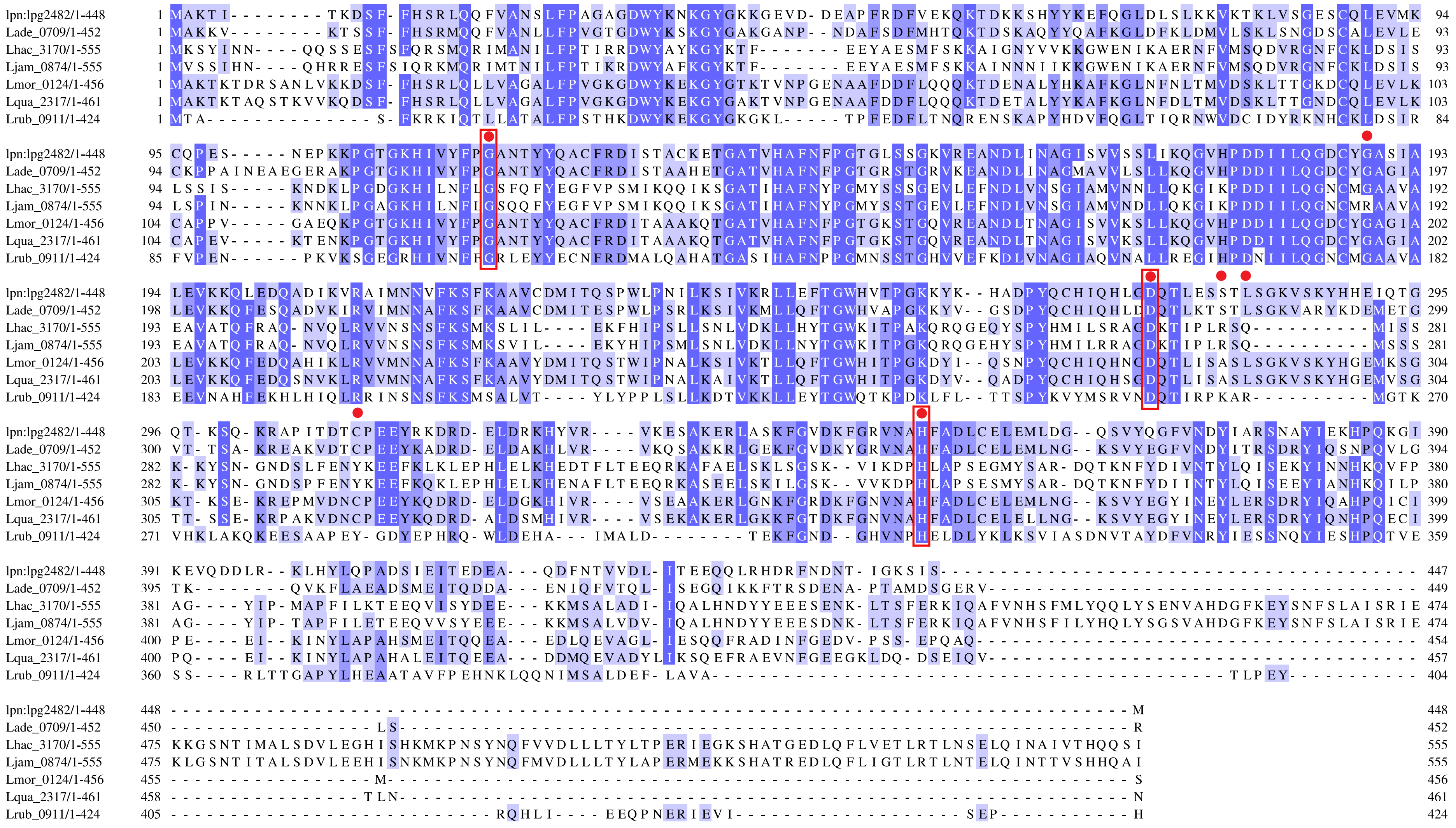

Figure S1: Random mutagenesis targets conserved residues in SdbB orthologs from seven *Legionella* species. The sequences of SdbB orthologs identified by Burstein *et al.* (Nat Genet 48(2): 167-175, 2016) were aligned using T-coffee, visualized with Jalview and coloured by % identity. The missense mutations identified by the random mutagenesis screen are indicated with a red closed circle above the SdbB (Lpg2482) sequence. Three of the seven mutations target invariant residues: G116, D273 and H351 shown in red boxes.
