# Supplementary material for: A random mutagenesis screen enriched for missense mutations in bacterial effector proteins
Source: G3 (Bethesda). 2024 Jul 19;14(9):jkae158. doi: 10.1093/g3journal/jkae158 (PMC11373652; doi:10.1093/g3journal/jkae158)
Supplement: jkae158_Supplementary_Data [file jkae158_supplementary_data.zip › Figure_S2_G3-2024-405229.pdf]

```
SidB   1 MAKIYNAPKPKYSGWEWFKFIAIRTVFPPVLLWDLIKIGANKLLGEWVSGLVLPAQNENF
SdbB   1 MAKTITKDSFFHSRLQ--QFVA-NSLFPAG-------------AGDWYKNKGYGKKGE-V

SidB  61 DDLAISDDTVSNYNED------------D--LICEKHDVITHDGAHLDTFEVRHRSQESI
SdbB  44 DDEAPFRDFVEKQKTDKKSHYYKEFQGLDLSLKKVKTKLVSGESCQLEVMKCQPESNEPK

SidB 107 DPKYQKYIINLVGNGMCYEHIIDDIKEDSKALKANVIGFNLRGVGQSTGKAKSSEDLVAD
SdbB 104 KPGTGKHIVYFPGANTYYQACFRDISTACKETGATVHAFNFPGTGLSSGKVREANDLINA

                              GxS/CxG
SidB 167 GIAQVQRLLDQGVSPQNITLKGHSLGAGVASLVAQHFHQLGQ-PINLFNSRSFSTITNFL
SdbB 164 GISVVSSLIKQGVHPDDIILQGDCYGASIALEVKKQLEDQADIKVRAIMNNVFKSFKAAV

SidB 226 VGHMRLERDEIGRAIGHKDSTVGTILGWLAKPFIKFGVALAKWEINAGSAFKSVPEAYKD
SdbB 224 C----------------DMITQSPWLPNILKSIVKRLLEFTGWHVTPGKKYKHA-DPYQC

SidB 286 YIVVRSRKEIRGERIDDAVIPHYASIHKELASERHKKKAEIDEEIANLDDIIRKADPLAK
SdbB 267 HIQHLGDQTLESSTLSGKVSKYHHEIQT--GQTKSQKRAPITDTC--PEEYRKDRDELDR

SidB 346 PGLANARDALVQAREKIKSDRKMETDVQYANGHNSDWNALHNRSGKSA-QTFFREFVQRT
SdbB 323 KHYVRVKE---SAKERLASKFGVD-KFGRVNAHFADLCELEMLDGQSVYQGFVNDYIARS

SidB 405 EA---DHAVKSIPEIN--------------------------------------------
SdbB 379 NAYIEKHPQKGIKEVQDDLRKLHYLQPADSIEITEDEAQDFNTVVDLITEEQQLRHDRFN

SidB     ----------
SdbB 439 DNTIGKSISM
```

**Figure S2: SidB and SdbB amino acid sequence alignment**. The amino acid sequences of SidB and SdbB were aligned with T-coffee and visualized with Boxshade, where identical residues are shown in black and similar residues in grey. The active site motif GxS/CxG predicted by NCBI conserved domain search (Marchler-Bauer A et al., Nucleic Acids Res.45(D)200-3, 2017) for SidB is indicated, suggesting that SdbB C187 is part of the active site catalytic triad.
