# Supplementary material for: A random mutagenesis screen enriched for missense mutations in bacterial effector proteins
Source: G3 (Bethesda). 2024 Jul 19;14(9):jkae158. doi: 10.1093/g3journal/jkae158 (PMC11373652; doi:10.1093/g3journal/jkae158)
Supplement: jkae158_Supplementary_Data [file jkae158_supplementary_data.zip › Figure_S3_G3-2024-405229.pdf]

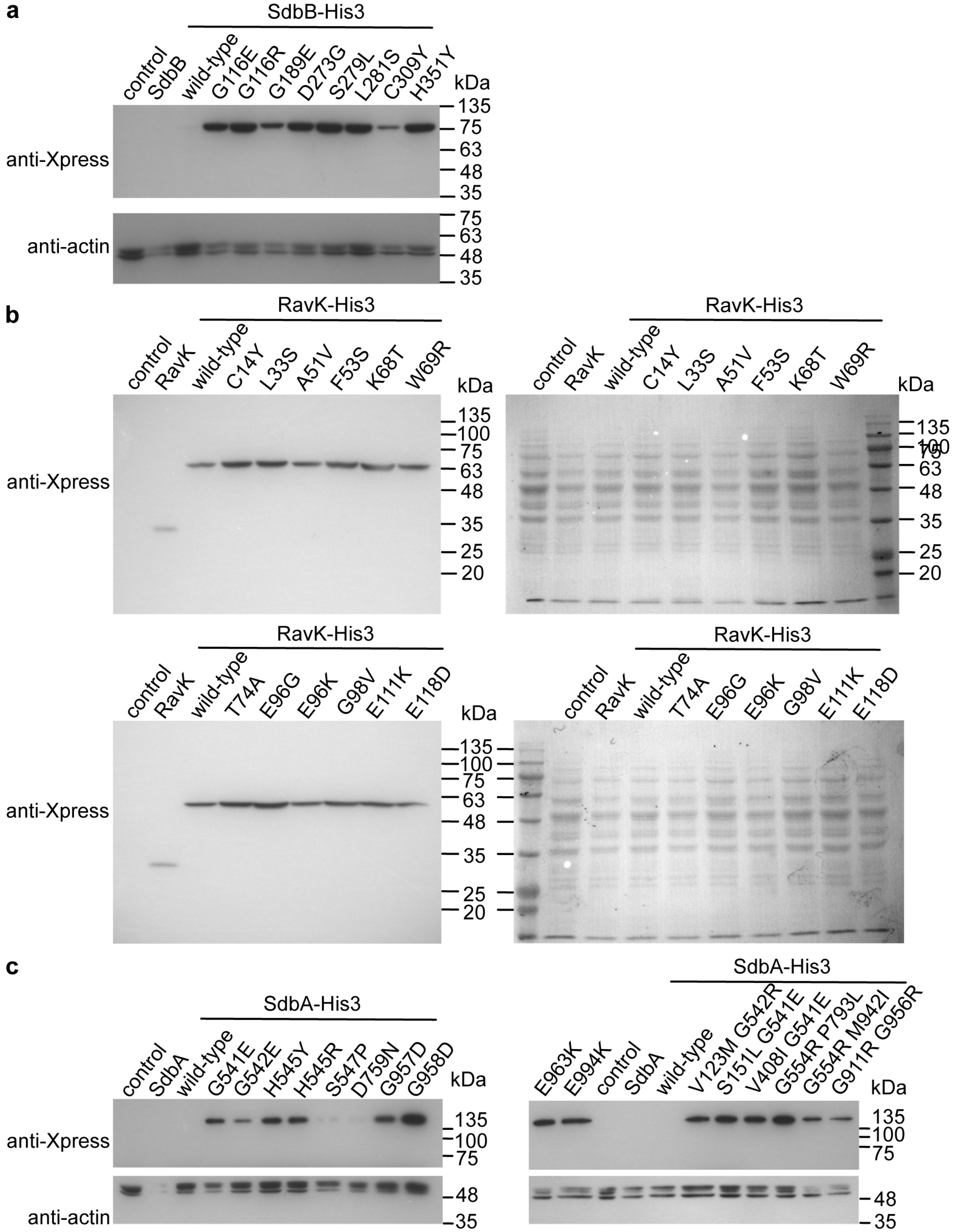

Figure S3. Expression of *effector-HIS3* wild-type and loss-of-function mutants. Yeast strains carrying pYES2NT/A effector-HIS3 mutant clones were grown overnight in SD-Ura/gluc, washed and diluted in SD-Ura/gal and grown for 6 h. Samples were analyzed by SDS-PAGE and western blot using the anti-Xpress antibody for the effectors and anti-actin as a loading control for SdbB and SdbA. **a)** Expression of Xpress-tagged SdbB, SdbB-His3 wild-type and mutants. Wild-type SdbB and SdbB-His3 are not detectable, but the loss-of-function mutants are. SdbB-His3 C309 has a lower expression level, but higher than the wild-type SdbB. **b)** Expression of Xpress-tagged RavK, RavK-His3 wild-type and mutants. Wild-type and mutant RavK can be detected in all samples. As RavK is an actin protease, the Ponceau S stain is shown as a loading control. **c)** Expression of Xpress-tagged SdbA, SdbA-His3 wild-type and mutants. Wild-type SdbA and SdbA-His3 are not detectable, but the loss-of-function mutants are. Clones with missense mutations S547P and D759N have a lower expression level, but higher than wild-type SdbA-His3.
