# Supplementary material for: A random mutagenesis screen enriched for missense mutations in bacterial effector proteins
Source: G3 (Bethesda). 2024 Jul 19;14(9):jkae158. doi: 10.1093/g3journal/jkae158 (PMC11373652; doi:10.1093/g3journal/jkae158)
Supplement: jkae158_Supplementary_Data [file jkae158_supplementary_data.zip › Figure_S5_G3-2024-405229.pdf]

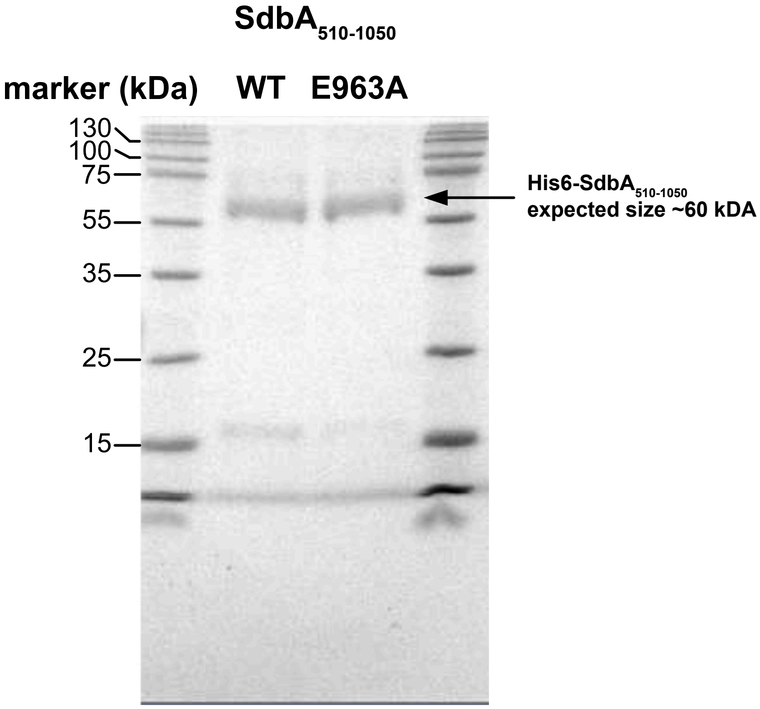

Figure S5: Purified SdbA$_{510-1050}$ wild-type and E963A.
The purity of the SdbA fragments used in the UDP-Glo Glycosyltransferase assay was assessed by SDS-PAGE and vizualized by Coomassie staining.
